# Using Massive Vehicle Positioning Data to Improve Control and Planning of Public Road Transport

**DOI:** 10.3390/s140407342

**Published:** 2014-04-23

**Authors:** Gabino Padrón, Carmelo R. García, A. Quesada-Arencibia, Francisco Alayón, Ricardo Pérez

**Affiliations:** Institute for Cybernetics, University of Las Palmas de Gran Canaria, Campus de Tafira, Las Palmas de Gran Canaria, 35017 Las Palmas, Spain, E-Mails: gpadron@dis.ulpgc.es (G.P.); aquesada@dis.ulpgc.es (A.Q.-A.); falayon@dis.ulpgc.es (F.A.); rperez@dis.ulpgc.es (R.P.)

**Keywords:** mobile positioning systems, automated data collection systems, intelligent transportation systems, pattern recognition

## Abstract

This study describes a system for the automatic recording of positioning data for public transport vehicles used on roads. With the data provided by this system, transportation-regulatory authorities can control, verify and improve the routes that vehicles use, while also providing new data to improve the representation of the transportation network and providing new services in the context of intelligent metropolitan areas. The system is executed autonomously in the vehicles, by recording their massive positioning data and transferring them to remote data banks for subsequent processing. To illustrate the utility of the system, we present a case of application that consists of identifying the points at which vehicles stop systematically, which may be points of scheduled stops or points at which traffic signals or road topology force the vehicle to stop. This identification is performed using pattern recognition techniques. The system has been applied under real operating conditions, providing the results discussed in the present study.

## 1. Introduction

Entities responsible for the regulation of public transportation on roads need data that allow them to verify that the operations of transportation companies are executed according to plan. Thus, these agencies frequently request the data from the companies in charge of providing this service that includes trip starting time, time history of stops, frequency and trips performed. In many cases, these data are difficult to provide because the companies do not have the necessary technology to acquire them. Thus, the data must be recorded manually, which is susceptible to errors. Authorities not only request these data for supervisory reasons but also use them to improve the planning of operations, for example, optimizing frequency, adjusting stop schedules to actual needs, improving infrastructure design of the transportation network, modifying the locations of stops, and improving stop infrastructure as a function of their use by travelers. The present article describes a system that allows regulatory agencies to perform these functions. The amount of data obtained by the system is massive, and the techniques used to manage the data are inspired by systems of ubiquitous data management.

The following section states the objectives and requirements of the system. The third section describes various studies related to the objective of defining the challenges and the novel nature of the proposal. The description of the system is addressed in the fourth section. The fifth section presents a practical application of the system. The main conclusions are stated in the last section.

## 2. Objectives of the System

The following are among the different responsibilities of public transportation regulatory agencies: control and verification of the activity of public transportation companies, improvement of the transportation network and improvement of services. To perform these tasks, authorities traditionally request information from transportation operators. However, it is not always easy for the transportation operators to provide this information due to the need to manage large volumes of data and because these companies may not possess the required technologies to satisfy these requests; thus, they have to employ manual methods of data collection that may result in errors [1]. The system presented in this study was conceived to obtain these data directly from vehicles belonging to a public road transportation fleet. When the problem to be solved involves management of large volumes of data, techniques to transmit and automatically process these data are needed. To that end, another challenge that needs to be overcome by the system consists of the intelligent management of large volumes of data using communication infrastructure. This context of intelligent data management involves acquiring, transmitting and processing data in an autonomous and transparent manner. For automatic processing of the data, the system suggests the use of standard data models that facilitate the interoperability of the data and the use of techniques that allow for obtaining useful information from the data. To obtain useful information from large volumes of data, we suggest the use of pattern recognition techniques. These patterns are associated with entities that are considered in the data models used to represent the transportation network. One of the basic entities used by the public transportation data models is the set of points at which vehicles make systematic stops, to either to pick up or drop off travelers or because there are traffic signals or elements on the road that require the vehicles to stop. Thus, the suggested system must provide data that allow for verification that the points where stops occur are those where the stops are planned to occur (stops or stations) and obtain the frequency of use of these points by travelers. In addition, the data provided by the system should help agencies to identify the points where unscheduled stops occur frequently. This information aids in estimating the duration of routes in a more realistic manner and in facilitating the compliance of the schedules published for users, an important aspect of the quality of the public transportation service [2].

In general terms, this study has four objectives, all of which are very important in the context of public transportation on roads. The first objective is to provide data that facilitates the quality control of the service. The second objective consists of acquiring data that allow for the improvement of planning by adjusting it to the needs of travelers. The third objective is to share vehicle information with the infrastructure of urban areas to facilitate the improvement of quality of life in cities and urban environments. The fourth objective is focused on the improvement of driving safety, providing useful information to the driver and automating tasks for him/her to be focused on driving the vehicle; ultimately, the point is to enable intelligent environments for vehicles.

To fulfill the stated objectives, a system is required that possesses the different elements (sensors, means for storage and processing, computation units, *etc.*) that provide the required data. The most important requirement of this system is preventing the system from interfering with the operation of all the devices installed in the vehicle and safe operation by the driver; therefore, it must work autonomously and in a transparent manner.

Related to the objectives described above, we can stand out three applications in which the results presented in this work are very useful:

1. To improve the representation of the transport network. As we have mentioned before, the applied method allows the detection of different points connected to positions that are not usually modelled in the representation graph of the transport networks; generally these are points where the vehicle that is carrying out the trip does not have the right of way, for example, in stops signals, traffic lights, yield signals or crosswalks. Moreover, the method allows the inclusion or elimination of these points dynamically, that is, if we detect singular points statistically where the vehicle stops, we applied the method so that we can determine what type of point it is. It is feasible to add or delete points to the graph according to their appearance in terms of cumulative statistics that involves applying the proposed method.
2. To improve the management of the infrastructure used in the maintenance of the stops. Applying the proposed method not only helps to detect where the vehicle stops and the corresponding points in the scheme used to model the network, but also according to the frequency that vehicles stops along the trip, it is possible to define an objective criterion that helps authority to define the infrastructure each stops should be provided with, both in material and maintenance.
3. To monitor compliance of planning. Transport authorities require an effective way to know the extent to which operators of the transport services comply with the contract and to what extent such compliance meets the demand of users. The proposed method allows the authorities to verify both, the vehicle routes and the frequency of each route.

## 3. Related Research Studies

Both transportation companies and regulatory authorities are interested in acquiring the information necessary to verify that public transportation services are provided according to plan to optimize their operations and improve the quality of the service they provide. According to Furth [3], this information is obtained from four different sources of data: surveys of travelers, rate of use records obtained from the vehicles, automatic passenger counting (APC) systems and automatic vehicle location (AVL) systems. Different studies related to each of these techniques for obtaining data are described next.

Surveys of passengers are a tool that is frequently used by companies and regulatory agencies to collect information about the use, preferences and assessments of public transportation users. While obtaining information in this manner is expensive, regulatory agencies regularly resort to surveys because they are very useful and precise [4].

According to Ryus *et al.* [5], the use of rate-of-use records obtained from vehicles has two associated problems: first, their high cost because they require human intervention, which leads to a reduced amount of data being collected as a result, and second, the possibility of errors in the data collected or in their transcription. In general, onboard devices that provide these data are driver consoles and reading/writing terminals of magnetic or intelligent cards.

APC systems are based on the use of different types of sensors, such as infrared light [6] and acoustic [7], optical [8] and even Bluetooth technology [9] to detect the presence of travelers in the vehicle. These sensors are usually installed in the entry and exit points of the vehicle. These systems are associated with the following problems: the information that can be obtained from them is limited, e.g., they do not provide data on the position of the vehicle and can contain errors due to incorrect operation of the hardware devices or an erroneous assessment of the presence of a passenger in the vehicle. In addition, depending on the technology being used, these systems require systematic maintenance activities such as recalibration of the sensors.

AVL systems are based on the use of different technologies, such as odometers [10], RFID [11] and GPS. These technologies can detect the presence of the vehicle at a specific point, for example, infrared markers, or locate the position of a vehicle at any moment in time, for example, GPS. These systems also have problems: first, they provide no data on the occupation of the vehicle, and second, systems that continually provide the position of the vehicle have a lack of precision. For example, a standard GPS can produce positions with an error radius of 100 m, and different techniques are required to reduce errors to 10 m. Two examples of these techniques are differential GPS, which requires constant communication with a remote place to receive the position corrections, and the technique proposed by Peyraud [12] for urban areas in which satellite signals are low quality that is based on image processing. Another problem associated with these systems is that for the information to be useful, the data must be recorded in a continuous manner.

The previous paragraphs cited research works that describe different techniques and technologies used to obtain useful data to aid in the control and improvement of public transportation for travelers. The following paragraphs will describe the studies that address what types of data are needed and in what quantity such that they can be useful for different agencies and companies, in other words, for them to be interoperable.

Planning of a public road transportation system for travelers involves determining in an optimal manner, if possible, a plan for routes, frequencies, schedules, personnel and fleet allocations. When optimizing a public transportation system for travelers, two conflicting objectives are primarily considered: maximizing the quality of the service versus maximizing the economic benefit. One method of reaching the objective of maximizing the benefit is through optimal allocation of the fleet and resources (vehicle and drivers). This approach has been the subject of multiple studies in the context of various disciplines. Primarily, the different techniques consist of modeling a classical combinatorial optimization problem and integer linear programming, and in many cases, the solution obtained is very close to the exact solution. Another way to reach the previous objectives is through the design and optimization of routes and frequencies. This approach has been less studied because it addresses a complex problem with a high computation cost that requires a large volume and different types of entry data. For example, Baaj and Mahmassnai [13] suggested a method with the objective of minimizing total passenger transfer types as well as the size of the fleets required. In another example, Israeli and Ceder [14] suggested a model with the objective of also minimizing transfer times and the fleet size. Lovell [15] suggests a model to calculate route frequencies. Finally, another approach based on rules is proposed by Barra *et al.* [16]. All of these models require a large amount of dynamic data (the data vary depending on the time of day and time of year) and of different types (transportation network geographic data, vehicle location, passenger movement, *etc.*), which must be obtained by turning to different sources of data.

## 4. Description of the System

**Figure 1.**
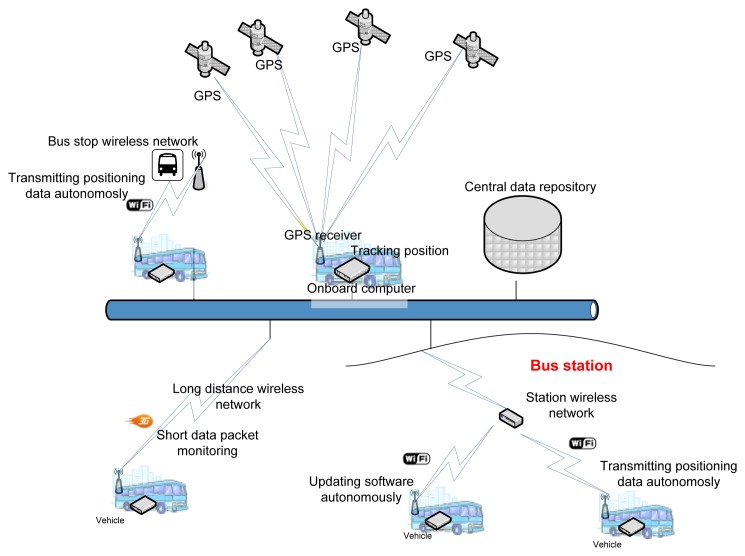
General view of the system.

The system illustrated above is an AVL system. The system obtains data and transfers it autonomously without interfering with the operation of the rest of the systems installed in the vehicle. Figure 1 presents a general schematic of the system. This system is structured on three basic components: the onboard computer, the positioning system and the data communication system.

The system only uses positioning data because the sensors available in a vehicle, for example, those that detect open/closed doors, vary from one producer to the next. In addition, when these sensors are available, they are not always accessible for devices that are different from those provided by the vehicle manufacturer.

### 4.1. The Onboard Computer (OBC)

The onboard computer is responsible for obtaining, processing and storing all the relevant data provided by the positioning system and controlling the transfer of data to data storage units in the transportation network. The computer is able to operate in adverse environmental conditions (temperature and vibrations), has a small size and has sufficient computation and storage capacity to execute all the processes required in this intelligent system. The more relevant characteristics of the computer used by the system are as follows: reduced dimensions (11.5 × 10.1 × 2.7 cm), a weight not greater than 330 grams, a 2 GHz CPU, a 2 GB DDR2 memory, power consumption not greater than 10 W, an operating temperature that varies between 0 and 70 °C and a voltage range between 8 and 14 V. These parameters facilitate its installation given that its voltage levels can be provided by a conventional vehicle electric system (which provides voltage of up to 25 V). Finally, the computer is equipped with a Wi-Fi communication interphase and four USB 2.0 ports. This computer has been fitted with a 64 GB compatible 2.5 ″ solid-state hard drive (SSD). A solid-state drive is used because these storage devices can tolerate more intense vibration levels than conventional magnetic devices and do not increase the ambient temperature.

### 4.2. The Vehicle Positioning System (VPS)

The system uses a GPS receptor. The data obtained are the vehicle's position (latitude, longitude and elevation), velocity, the quality of readings, which is a function of the satellite coverage, and the instant in time at which the universal time coordinates (UTC) were measured. It is important to note that GPS units are commercially available and provide geographical position readings with an error that is a function of different factors: climate conditions at the time of data acquisition, terrestrial curvature radius and the presence of random selective error. In general, assuming there is no random selective error, the maximum positioning error for GPS is 100 m. The connection with the OBC is made through an asynchronous interphase with the following communication parameters:

- Baud rate: 9,600.
- Data Bits: 8.
- Stop Bits: 1.
- Handshake: no.
- Parity: no.

The protocol used for communication between the GPS and the OBC uses a data packet with the following structure:

>ABB{C}[;ID=DDDD][;*FF]<,

where:
>:Start Indictor of the data packetA:Message qualifierBB:Message identifierC:Character chainID=DDDD:Vehicle identifier (optional)FF:Checksum (optional){x}:Means it can occur any number of times[x]:Means it can occur only once

The GPS receiver sends a packet every second with the previously described structure, for which the field C is constituted by a message that has the following structure:

AAAAA.BBBCCCDDDDDDDEEEEFFFFFFFGGGGGGGHHIIIJKKKKLMMMNOOPPQQPPQQ…PPQQRRRRRRRRRRST,

where:
C1:AAAAA.BBBGPS date and timeC2:CCCDDDDDDDLatitude expressed in degreesC3:EEEEFFFFFFFLongitude expressed in degreesC4:GGGGGGGHHElevation expressed in feetC5:IIIJVertical velocityC6:KKKKLHorizontal velocityC7:MMMNHeadingC8:OONumber of satellites used in the measurementC9:PPIdentifier of the satellite constellationC10:QQIODEC11:RRRRRRRRRRReservedC12:SSource of the readingC13:TAge of the reading

The data structure used to store each position reading is called the *Position Record (PR)* and has the structure shown in Table 1:

All the *PR* data are stored in a table in the system database called the *Positioning Table (PT)*.

### 4.3. The Communications System

This element allows the OBC to communicate with other external systems through wireless communication infrastructures: 3G/GPRS/GSM and Wi-Fi. The 3G/GPRS/GSM infrastructure allows long-distance communication and is used for communication of technical alarms for any anomaly in the OBC or in the VPS. This communication is performed through short data packets. Wi-Fi communication is used to transfer data from the vehicle and the control center. Wi-Fi is also used to automatically update the onboard computer software. The Wi-Fi infrastructure, which allows vehicles to transfer data autonomously, consists of a set of access points located at points of the transportation network along which vehicles frequently pass and where they also remain static during significant periods of time: stations, stops at the beginning of the route, garages and shops. To perform intelligent communication and optimize the consumption of energy when executing data transfers, the system uses a geographical database that contains locations of Wi-Fi coverage points. Thus, the system uses the vehicle positioning provided by the VPS to determine when it can establish a connection to transfer data.

### 4.4. Data Management

Considering that the system stores the vehicle's position every second, from the viewpoint of data management, the system represents a case of a mobile system for massive data management. To guarantee the integrity of the data, the system uses the principles of data management in ubiquitous environments. According to Perich [17], this type of system is characterized by four properties. The first one is the capacity to operate in environments in which the number of data spaces and applications that access those data spaces is dynamic. The second property is the capacity to operate with different catalogues and data schemes. The third property is necessary due to the high risk of data inconsistency that exists in ubiquitous environments, caused by the spontaneity of connections and disconnections of the applications that can access them, and consists of providing mechanisms that resolve these possible inconsistencies. The fourth property is that collaboration mechanisms must exist between the applications to facilitate access to the data.

In our case, these challenges are resolved through a collaborative model of agents that obtain the data provided by the different devices of the previously mentioned systems [18]. The conceptual model and its implementation in the form of a database are described in García [19]. Primarily, all relevant events that occur in the vehicle are represented by a set of data produced by devices (sensors, driver console, card readers, *etc.*). The amount of data and the structure of this set of data vary and depend on the type of event represented. Ontology is used to guarantee the interoperability of the data and is implemented through a catalog and data scheme based on the conceptual model for public transportation TRANSMODEL [20].

## 5. Use Case

This section describes a system's use case. The system is used to obtain points on the route of a vehicle at which it systematically stops. This information is useful to regulatory agencies because they can:

- Verify that the stops used by transportation company vehicles are located at the positions that were agreed upon with transportation companies.
- Determine the frequency of use of each of the stops. This information is useful to adequately define the locations of the tops in the transportation network and determine how they are outfitted.
- Improve knowledge about the transportation network by incorporating new entities that affect the duration of vehicle routes. Specifically, these new entities are points on the routes that are not scheduled to be used for passenger pick-up or drop-off (stops and stations) but are where vehicles systematically stop or reduce their speed, therefore affecting the duration of the trips; these are also of interest when attempting to obtain information about the state of traffic on the roads that make up the transportation network.

This case of application describes how the system is able to identify points at which the vehicle systematically stops. These points can be of two types:

- Points of scheduled stops (PP, for its initials in Spanish). Points at which the vehicle must stop according to plan: stops and stations.
- Singular points (SP). These points are locations where the vehicle systematically stops due to the presence of traffic signals (traffic lights, pedestrian crosswalks, stop signs, *etc.*) or changes in road topology (road narrowing, roundabouts, intersection with another road without having the right-of-way, *etc.*).

The method used is capable of identifying SP type points in a route while only making use of the data provided by the system. Using only GPS readings, the precision of these readings must be considered, which as was mentioned before, can have a maximum error of 100 m. Therefore, the technique described has a restriction; the distance between two consecutive systematic stop points must be greater than 100 m. The technique is based on executing the following steps:

(1). Select the vehicle positioning data sample.
(2). Filter erroneous or irrelevant GPS readings.
(3). Classification:
  (3.1.) Identify the set of scheduled stop locations in the route (PP).
  (3.2.) Identify the set of locations at which the vehicle systematically stops without the stops being systematic stop locations (SP).

**Figure 2.**
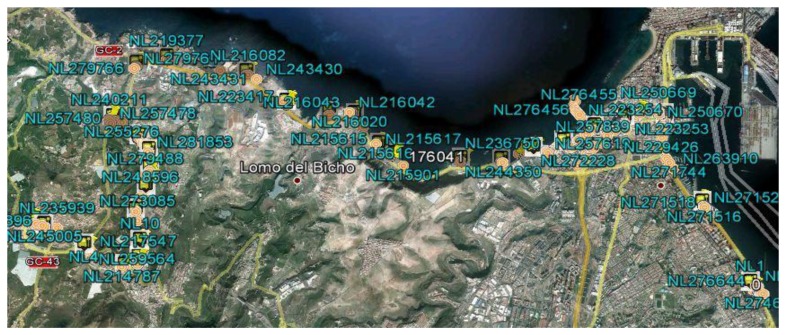
Aerial view of the geographic area of the route being studied. The green tags represent vehicle position readings with a velocity of zero. The orange markings represent the centroids of the groups of readings with a velocity of zero. The yellow icons represent scheduled bus stops of the route.

A description of each of these steps follows, including the results obtained when applying this technique to an interurban route traveled by a vehicle selected from the fleet, called the laboratory vehicle. The route being studied has an approximate length of 23 km and duration of 40 min. The route passes through urban areas with high traffic density and non-urban areas with a more fluid traffic. The explanation will be complemented with aerial photographs of the sample route provided by the Google Earth service (Figure 2).

Step 1: **Selection of the Vehicle Positioning Data Sample**
  As mentioned previously, the onboard system makes periodic measurements every second of the vehicle position and stores these measurements in the PT table described in the section about data management. To obtain the positioning reading sample, a significant period of time is established, and all the records obtained in that period are extracted from the PT table. A period of 3 months was established for the route being studied, resulting in a sample of 7,862,400 positioning readings.
Step 2: **Filtering Erroneous or Irrelevant GPS Readings**
  The records in the PT table, the PR record described previously, store the vehicle positions; however, the GPS receptor does not always provide a reliable reading, which is why it is necessary to filter the PR records that contain erroneous or unreliable readings. The detection of unreliable GPS readings is based on the use of fields 7 and 8 of the PR record. Field 7 has a length of 1 byte and can take the following values:
02D reading: Only the latitude and longitude are provided, and the elevation reading is not provided.13D reading: The latitude, longitude and elevation are provided.22D reading in a differential GPS configuration (DGPS).33D reading in a differential GPS configuration (DGPS).6DR.8Degraded DR.9Unknown source.
  Field 8 informs us about the age of the reading provided:
0Reading unavailable.1Old reading: obtained more than 10 s ago.2Recent reading: obtained less than 10 s ago.
  Because the GPS system is not differential, filtering of GPS readings is performed by eliminating all the readings that do not include values 1 and 2 in fields 7 and 8, respectively. In addition to the samples discarded for being invalid or unreliable, irrelevant readings will also be eliminated to solve the problem. As the problem lies in identifying the points at which the vehicles systematically stop, the samples associated with positions at which the vehicle was moving are eliminated, *i.e.*, readings that have a velocity not equal to zero (fields 5 and 6). Once these readings are eliminated, the resulting set of vehicle positions with zero velocity is called the Z-set. In the route being studied, applying this filter to the set of readings obtained in step 1 yields a Z-set of approximately 70,000 reliable position readings that indicate that the vehicle had a velocity of zero. When placing these readings on an aerial photograph of the geographic area containing the route being studied, one can see that the majority of the readings are grouped. These groups are associated with the locations of scheduled stops and the locations of unscheduled stops, the latter being points SP, and the objective of the method described is to identify them (Figure 2). When interpreting this graphical representation, we must note that a conventional non-differential GPS unit provides readings with a maximum margin of error of 100 m and that the stops in the transportation network have varying lengths. For the case of the route being studied, the average length of stops is 15 m.
Step 3: **Classification of the Points**
  The identification method is based on the following hypothesis: “If we consider the set of reliable and relevant readings of the problem, that is, the Z-set, the readings obtained for points where the vehicle systematically stops will have a high frequency compared with those of points at which the vehicle stops sporadically. In addition, the readings obtained at points of systematic stops must be grouped, with the number of groups being equal to the number of existing systematic stops along the route”.
  In accordance with the previous hypothesis, if we were to calculate the frequency histogram of the Z-set, we would observe the following behavior: there will be a high frequency of readings at the points of the route at which the vehicle stops systematically (PP-set and SP-set), whereas the readings associated with the positions around points at which the stop is not systematic will occur at a lower frequency. In addition, the readings in the Z-set will tend to form concentrated clusters when the vehicle is at one of the stops scheduled along the route. Considering this interpretation, the method used to identify the points of types PP and SP on the route of a vehicle is based on the detection of these groups and subsequent classification into two categories: scheduled stop points (PP) and singular points (SP). The technique used is a recursive one in which the K-means classification algorithm is applied in each step to a subset of readings of the Z-set. In the first iteration, the K-means algorithm is applied to the entire Z-set, using as the initial solution the set of scheduled stops (PP-set) that have been identified by their positions. The number of stops along the route is denominated N. Once the K-means algorithm is applied, it will converge on a solution with many clusters equal to N. In Figure 2, the orange markings represent the centroids of the clusters, and the yellow markings represent the stops along the route being studied. There are 28 clusters represented by their centroids and 28 scheduled stops. The positions of the majority of the centroids are close to the positions of the points representing scheduled stops.

The following step involves identifying each of these clusters as a cluster associated to a point of the PP set or a cluster associated with a point in the SP set. The identification technique used is based on the use of two parameters:

- The distance between the centroids of each of the clusters and the closest point representing a scheduled stop. The distance used is the Euclidean distance.
- The scatter of the readings associated to each cluster relative to its centroid. The scatter measure used is the variance.

**Figure 3.**
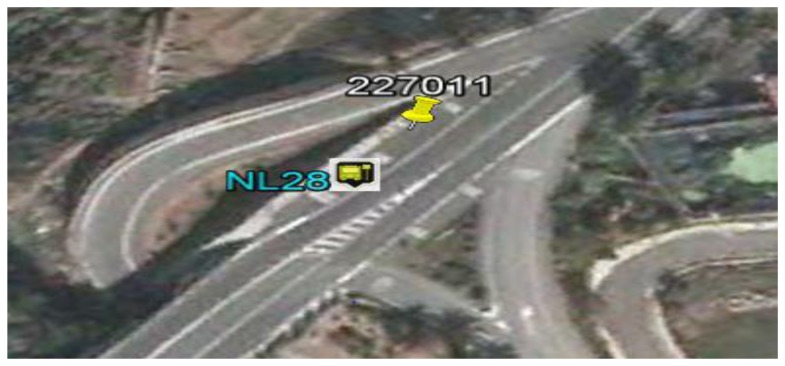
Aerial view of a cluster of type G1. Tag NL28 represents the centroid of the group of readings obtained when the vehicle stopped. Tag 227011 represents the position of the stop.

**Figure 4.**
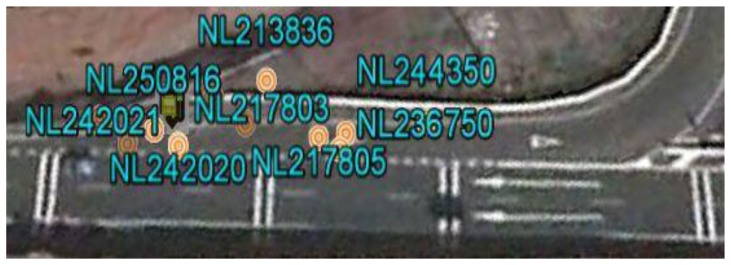
Aerial view of a type G2 cluster. The orange markings represent position readings of the vehicle with zero velocity at a location where no stop is programmed. The vehicle stops because it reaches a road with greater circulation priority. The yellow marking is the centroid of the cluster.

**Figure 5.**
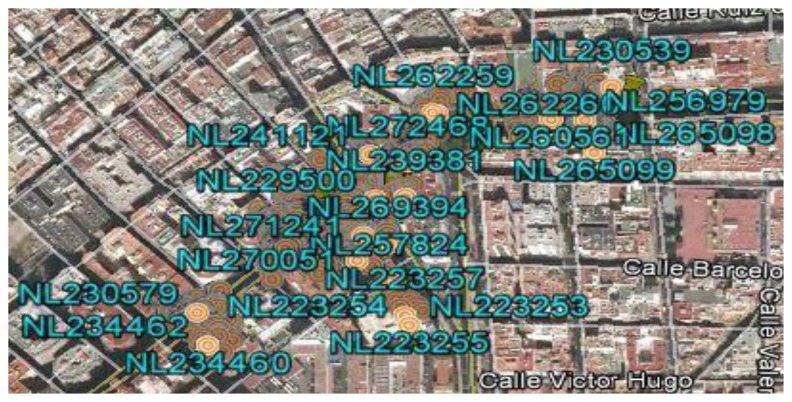
Cluster of type G3. The orange markings are position readings of the vehicle with a velocity of zero. There are two scheduled stop points in the area shown; however, all the readings have been grouped into one cluster, with a centroid at a distance of more than 15 m from the two scheduled stops.

The rules used to identify the clusters are as follows:

- **G1: Cluster associated with the PP-set (point of a scheduled stop).** If the cluster has a centroid very close to a point in the PP set and the scatter of the readings belonging to the cluster is low, then it is a set of readings associated with a scheduled stop of the vehicle. Because each PP point represents an area reserved for the vehicle to stop, a centroid is considered to be very close to a PP point if the distance between them is not more than 15 m (average length of the area reserved for the vehicle to stop). Figure 3 presents a case of this type on the route being analyzed. An aerial photograph is presented of a stop along the route, where the point with tag NL28 is the centroid of the cluster obtained when applying the K-means algorithm and the point with tag 227011 is the theoretical position of the stop.
- **G2: Cluster associated with a point in the SP-set (singular point on the route).** If the centroid of the cluster is not close to any point in the PP-set and the scatter of the readings belonging to the cluster is low, then it is a set of readings associated with a singular point in the route, with the centroid being the position that represents the point SP being identified. Figure 4 presents a case of this type along the sample route. An aerial photograph is presented of a point along the route at which the vehicle does not have the right of way; the circular points are readings of the cluster, and the green point is the centroid of the cluster obtained when applying the K-means algorithm.
- **G3: Unidentified cluster.** If the cluster exhibits high scatter in the readings, then it is a cluster with readings associated with more than one point at which the vehicle stops systematically (points in the PP-set and points in the SP-set). Figure 5 presents a case of a cluster of this type, and the high scatter of the readings is apparent.

For each cluster of type G3, a new iteration is performed, and each time, the K-means algorithm restricted to the points assigned to the cluster being studied is applied, taking as an initial approximation in the second and following iterations the points of type PP that are known, that belong to the cluster and the centroid of the cluster obtained. Thus, the points will be grouped on the new centroids, each of which is located around the PP and SP points of the cluster. The operation must be reiterated until a result is obtained for which the points are grouped at short distances from the centroids of their cluster. If the centroid of one of these clusters is very close (less than 15 m) to a point known as being of type PP, then the cluster represents readings of type PP. In contrast, if the centroid is not close to a point known to be of type PP, then the cluster is associated with a point of type SP that has been identified. Figure 5 presents a case of this type of cluster (G3) in a first execution. The method ends its iterations when all the clusters generated in the different levels of recursion are of type 1 or type 2.

**Figure 6.**
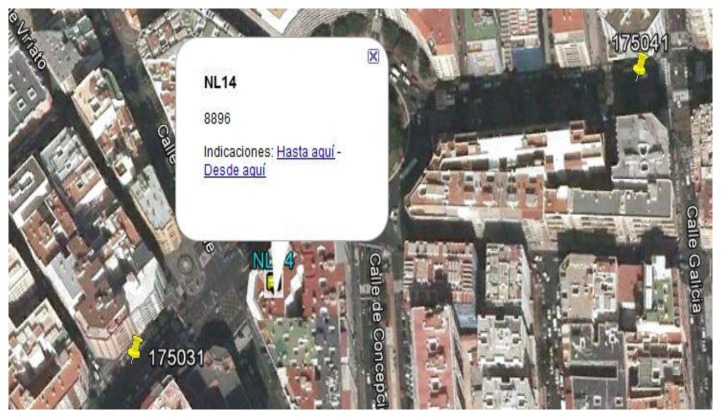
Close-up aerial view of the same cluster presented in Figure 5. Result of the first iteration of the method. Tags 175031 and 175041 are scheduled stop points, and tag NL14 is the centroid obtained in the first iteration of the method. The readings have been grouped in a single cluster because there is a roundabout between the two scheduled stop points where the vehicle stops.

**Figure 7.**
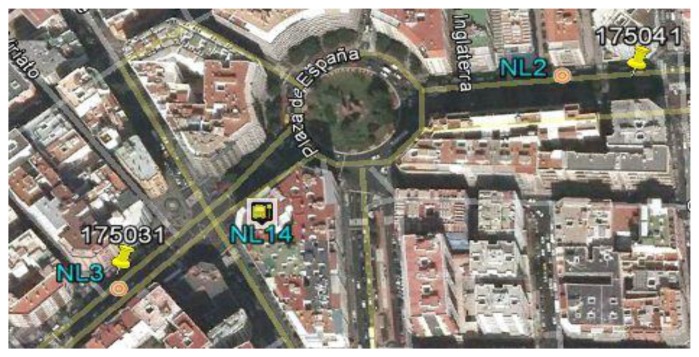
Result after applying a second iteration of the method to the cluster shown in Figures 5 and 6. The method obtains three clusters, two associated with the stops and another one associated with the singular point, which is a roundabout. As the scatter of the clusters is less than 15 m, the identification process is considered to be complete.

According to what has been discussed, the presence of a cluster without classifying implies a recursive invocation to the identification procedure. The unidentified cluster (type G3) is shown in Figure 5 and is made up of 8896 readings of the initial Z-set. Because of the first invocation, the method identifies a cluster with its centroid identified by tag NL14 located in the urban area and includes two points PP (points with tags 175031 and 175041) and a roundabout (Figure 6). Next, the method is applied recursively, restricted to the readings in this cluster, with the following initial parameters: form three clusters, and the initial approximations to their centroids are the points 175031, 175041 and NL14. When executing the second iteration, three clusters are obtained, represented by centroids NL2 of type G1, NL3 of type G1 and NL14 of type G2, with all the readings at a distance of less than 15 m from the centroid representing its class (Figure 7).

## 6. Conclusions

This paper has described an automatic vehicle positioning data collection system for public transportation on roads. The system was developed to help transportation regulatory agencies perform their objectives related to the control and improvement of public transportation. The system works autonomously, acquiring position readings for the vehicle and storing and transferring these readings automatically to remote data banks held by the regulatory authorities. In addition, the system works transparently and without interfering with the remaining systems installed in the vehicle. To attain its objectives, the system only uses position information provided by a GPS, without requiring data from the automatic passenger counting systems or rate-of-use systems used by transportation companies. Wi-Fi technology is used to transfer the positioning data and for software updates. 3G/GPRS/GSM technology is used to communicate data related to information on the state of the different elements of the system. Due to its structure and requirements, the system can be used in any type of public transportation vehicle that operates on roads.

To illustrate the utility of the system, we have presented a case study of its application. This practical application consists of the verification and identification of the locations at which the vehicles stop systematically along a route. This functionality allows the regulatory agency to verify that the locations where stops occur are those planned and agreed upon by transportation companies, verify the frequency of use of the stops used by the vehicles to adequately define the stops in the transportation network, and identify points that are not stops but at which the vehicles stop systematically due to traffic signals or the topology of the road on which the vehicles circulate. This identification would allow regulatory agencies improve the representation of the transportation network and design better routes. To perform this identification, K-means, the classic technique of pattern recognition, was used.

## Figures and Tables

**Table 1.** PR structure.

**Field Name and (Source Field in LNM Message)**	**Type and Size**
Universal Time Coordinated (C1)	Integer 4 bytes
Latitude (C2)	Float 4 bytes
Longitude (C3)	Float 4 bytes
Elevation (C4)	Float 4 bytes
Vertical velocity (C5)	Float 4 bytes
Horizontal velocity (C6)	Float 4 bytes
Source of the reading (C12)	Char 1 byte
Age of reading (C13)	Char 1 byte
Vehicle (On Board system parameter)	Integer 2 byte
